# Mitral Valve Infective Endocarditis Complicated by Aortic Root Abscess: A Rare and Fatal Progression

**DOI:** 10.7759/cureus.88610

**Published:** 2025-07-23

**Authors:** Esperance M Madera, Alla Adelkhanova, Mudassar Adeem, Bushra Ahmed, Rodolfo Aguilar-Guerrero, Beenish Zaman, Mohamed M Ali, Christine Lopez-yang, Alwin Thoms, Ammar Ahmed

**Affiliations:** 1 Internal Medicine, Mount Sinai Hospital, Chicago, USA; 2 Internal Medicine, Ross University School of Medicine, Florida, USA; 3 Critical Care Medicine, Mount Sinai Hospital, Chicago, USA

**Keywords:** aortic root abcess, infective endocarditis, mitral valve, septic shock, transthoracic and transesophageal echocardiography

## Abstract

Infective endocarditis (IE) is a life-threatening condition associated with high morbidity and mortality. Although aortic root abscesses are most commonly associated with aortic valve involvement, this case illustrates that mitral valve endocarditis, particularly in the presence of prosthetic material, can also lead to this rare and fatal complication. Prompt diagnosis and multidisciplinary coordination are critical to improving patient outcomes.

We report the case of a 69-year-old male with a history of coronary artery disease status post-coronary artery bypass grafting (CABG) and mitral valve annuloplasty, who was transferred from an outside facility for evaluation of a developing small bowel obstruction. Upon admission, he was found to be in septic shock and was admitted to the intensive care unit (ICU). Empiric broad-spectrum antibiotics were initiated, and blood cultures subsequently grew methicillin-sensitive *Staphylococcus aureus* (MSSA), prompting de-escalation to targeted therapy with gentamicin and cefazolin. Transthoracic echocardiography revealed vegetations on the mitral annuloplasty ring with concern for extension into the aorto-mitral curtain. Transesophageal echocardiography (TEE) confirmed the presence of an aortic root abscess.

Despite confirmation by TEE, cardiac magnetic resonance imaging/computed tomography (MRI/CT) was pursued to further delineate the extent of paravalvular involvement and to guide operative planning. However, surgical intervention was deferred due to persistent hemodynamic instability, multi-organ dysfunction, and an overall prohibitive surgical risk profile. The patient was transferred to a tertiary care center for further management but ultimately expired 36 days after diagnosis. His clinical course was marked by progressive septic shock requiring maximum vasopressor support and acute hypoxic respiratory failure necessitating intubation, underscoring the rapid progression and high lethality of the disease.

This case highlights a key learning point, which is that aortic root abscess, though classically associated with aortic valve IE, can occur as a complication of mitral valve endocarditis, particularly in patients with prosthetic devices. Early recognition, careful surgical risk stratification, and appropriate use of advanced imaging are essential. Further studies are needed to define optimal surgical timing, improve risk stratification models, and establish imaging protocols for high-risk patients with complex infective endocarditis.

## Introduction

Infective endocarditis (IE) is a relatively uncommon condition, with an estimated incidence of 3-10 cases per 100,000 person-years, but it carries significant morbidity and mortality, reaching up to 30% at 30 days if not promptly diagnosed and treated [1,2]. Among native valve infections, left-sided valves are more frequently involved, with the mitral valve affected more often than the aortic valve. The most common causative organisms are *Staphylococcus aureus* and *Streptococcus* species.

Given the wide variability in clinical presentation, diagnosis of IE relies on the modified Duke criteria, microbiologic data, and imaging, most notably transesophageal echocardiography (TEE), which remains the gold standard for detecting valvular vegetations and periannular complications such as abscesses [3]. Management often requires prolonged antimicrobial therapy, and up to 50% of patients require surgical intervention [4]. Indications for surgery include large vegetations, paravalvular abscess, embolic events, persistent bacteremia despite antibiotics, fungal infection, and moderate-to-severe valvular dysfunction [2].

An aortic root abscess (ARA) is a rare but life-threatening complication of IE, typically associated with aortic valve infection. The reported incidence of ARA among patients with IE ranges from 10% to 30%, with higher rates in prosthetic valve endocarditis [5]. ARAs most frequently result from *S. aureus* infections and may present with conduction abnormalities such as prolonged PR intervals or complete heart block.

Although ARAs are classically linked to aortic valve involvement, rare cases have been reported where infection extends from the mitral valve. This can occur due to the close anatomical proximity of the mitral annulus to the aorto-mitral curtain and fibrous trigone, allowing direct extension of infection into the aortic root. One large surgical case series reported only 1% of root abscesses originating from mitral valve infections, underscoring the rarity of this complication [6].

We present a unique case of mitral valve endocarditis complicated by the development of an aortic root abscess, highlighting the importance of early recognition, advanced imaging, and multidisciplinary management in atypical presentations of IE.

## Case presentation

History of present illness

A 69-year-old male with a history of end-stage renal disease on hemodialysis, diabetes mellitus, coronary artery disease status post-coronary artery bypass grafting (CABG), mitral valve annuloplasty, atrioventricular nodal reentrant tachycardia, and heart failure with reduced ejection fraction (HFrEF) presented to an outside facility (day 0) with three days of malaise, fatigue, and generalized weakness following a recent hemodialysis session.

Initial evaluation and transfer

On presentation, he was hypotensive (BP 85/69 mmHg), tachycardic (110 bpm), tachypneic (24/min), mildly febrile (T 99.9°F), and hypoxic (SpO₂ 84% on room air). Initial laboratory testing revealed leukocytosis (WBC 16.65 x10³/mcL, neutrophils 87.3%) and a lactate level of 1.7 mmol/L (Table 1). A chest X-ray demonstrated mild interstitial pulmonary edema, and an abdominal/pelvic CT raised concern for a developing small bowel obstruction (SBO). Blood cultures were drawn, and the patient was diagnosed with septic shock. He was started on empiric broad-spectrum antibiotics and IV fluids and transferred to our facility the following day (day 1) for further evaluation and surgical consultation.

Upon arrival, he remained hypotensive (BP 94/58 mmHg), tachycardic (106 bpm), and afebrile (T 99.1°F) and required 4 L/min of oxygen via nasal cannula to maintain SpO₂ at 98%. His leukocytosis worsened (WBC 17.1 x10³/mcL), and lactate increased to 2.2 mmol/L (Table 1). He was admitted directly to the ICU. General surgery was consulted on the same day and recommended nasogastric decompression, ultimately determining that the SBO was not clinically significant and did not require operative intervention.

**Table 1:** : Laboratory results from outside hospital and upon admission

Test	Outside Hospital Value	Admission Value	Reference Range
White blood cells (WBCs)	16.65 × 10³/mcL	17.1 × 10³/mcL	4.0 – 11.0 × 10³/mcL
Neutrophils	87.3%	87%	40 – 70% (typical range)
Lactate	1.7 mmol/L	2.2 mmol/L	0.5 – 2.0 mmol/L

The patient denied any recent dental procedures or surgeries. Social history was negative for tobacco, alcohol, or illicit drug use, and a urine drug screen was negative. He did not have a hemodialysis catheter; he was receiving dialysis via a mature left upper extremity arteriovenous (AV) fistula, which showed no signs of infection.

Blood cultures collected at the outside hospital became positive within 12 hours, indicating a high bacterial burden. Methicillin-sensitive *Staphylococcus aureus* (MSSA) was isolated. Susceptibility testing confirmed sensitivity to multiple antibiotics. Specifically, the patient was found to be susceptible to clindamycin at a concentration of ≤0.25 mcg/mL, erythromycin at ≤0.25 mcg/mL, gentamicin at ≤1 mcg/mL, and oxacillin at ≤0.25 mcg/mL. It also showed susceptibility to trimethoprim/sulfamethoxazole at a concentration of ≤0.5/9.5 mcg/mL. Additionally, susceptibility to vancomycin was confirmed, although the minimum inhibitory concentration (MIC) was not specified.

Based on these findings and in consultation with the infectious disease team, the patient was started on targeted antibiotic therapy with cefazolin and gentamicin. Repeat blood cultures obtained five days after initiating treatment were negative, indicating microbiologic clearance. Inflammatory markers showed a partial downward trend, supporting initial treatment efficacy.

**Figure 1:**
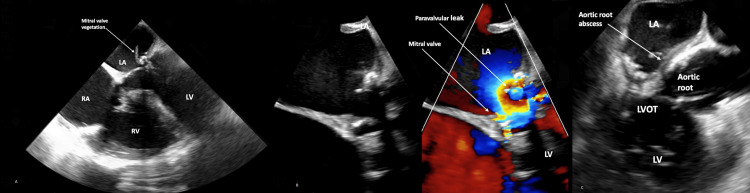
Transesophageal echocardiogram A. Mid-esophageal four-chamber view demonstrating a mobile, stalk-like filamentous vegetation attached to the mitral annuloplasty ring. B.  Mid-esophageal view of the mitral valve demonstrating a paravalvular leak. C. Transesophageal echocardiogram: mid-esophageal aortic valve long axis view demonstrating echolucency suspicious for aortic root abscess.

A transthoracic echocardiogram (TTE) showed an ejection fraction of 35-40% with diffuse hypokinesis. On day 2, transesophageal echocardiography (TEE) revealed mobile vegetations (up to 1.0 cm) on the mitral annuloplasty ring (Figure 1A), moderate paravalvular mitral regurgitation (Figure 1B), and an echo-lucent region adjacent to the aorto-mitral curtain consistent with a developing aortic root abscess (Figure 1C). Cardiac MRI/CT was not performed due to both logistical unavailability at our facility and the patient’s hemodynamic instability.

Despite initial stabilization, the patient required escalating vasopressor support with norepinephrine and vasopressin due to persistent hypotension.

Surgical evaluation and transfer

Cardiothoracic surgery was consulted, but operative intervention was deferred due to ongoing septic shock, multi-organ dysfunction, and high operative risk. He was transferred to a tertiary center on day 6 for further evaluation and possible advanced imaging. However, cardiac MRI was again deferred due to continued clinical instability. Coronary angiography performed at the tertiary center revealed severe three-vessel coronary artery disease, which, though not the primary contraindication, further contributed to the surgical team’s decision to forgo intervention due to poor overall prognosis.

Outcome

Despite ongoing supportive care, the patient’s condition deteriorated with acute hypoxic respiratory failure necessitating intubation and escalating vasopressor requirements. Given the poor surgical candidacy and limited likelihood of recovery, the decision was made to transition to comfort-focused care. The patient passed away on hospital day 36.

## Discussion

Infective endocarditis (IE) remains a challenging clinical entity, particularly in patients with prosthetic material or prior valve interventions. While aortic root abscess (ARA) is a known complication of aortic valve IE, its occurrence in the setting of mitral valve endocarditis is exceedingly rare. This case highlights a unique presentation of mitral valve prosthetic endocarditis complicated by ARA, underscoring the need for heightened clinical suspicion, especially in complex patients with prior cardiac surgery and multiple comorbidities.

The anatomical proximity of the mitral valve annulus to the aorto-mitral curtain and fibrous trigone offers a potential route for infection to extend from the mitral to the aortic root. The aorto-mitral curtain is a fibrous, avascular structure separating the left atrium from the left ventricular outflow tract, and it provides a vulnerable interface for infection spread, particularly in the setting of prosthetic material or annular calcification. Anguera et al. reported that only 1% of aortic root abscesses originate from mitral valve infection, typically involving the aorto-mitral curtain in patients with prior surgical repair [6].

Prosthetic valve endocarditis (PVE) poses unique diagnostic and therapeutic challenges. Prior mitral valve annuloplasty likely altered native annular anatomy and introduced acoustic shadowing, complicating imaging interpretation. While transesophageal echocardiography (TEE) remains the gold standard for detecting valvular abscesses and periannular extension [4], the presence of prosthetic material can reduce sensitivity. In our case, TEE revealed mobile vegetations, moderate paravalvular regurgitation, and a suspicious echo-lucent lesion adjacent to the aorto-mitral curtain, findings concerning developing abscess. Unfortunately, advanced imaging with cardiac MRI/CT was unavailable at our facility and was later deferred at the tertiary center due to the patient’s hemodynamic instability and respiratory failure.

Management of PVE complicated by ARA typically necessitates early surgical intervention, especially when complications such as abscess formation, heart block, or persistent bacteremia are present [3,7]. However, this patient had several major comorbidities, including end-stage renal disease (ESRD), diabetes mellitus, and heart failure with reduced ejection fraction (HFrEF). These factors significantly limited his physiological reserve and surgical candidacy. Although the patient’s blood cultures cleared on appropriate antibiotic therapy (cefazolin and gentamicin), his progressive multi-organ failure and high operative risk led to a decision to defer surgery both at the primary and tertiary institutions. While earlier surgery might have improved his prognosis, surgical delay was unavoidable due to the severity of his initial presentation and the need for hemodynamic stabilization. Coronary angiography at the tertiary center revealed severe three-vessel disease, which further increased operative risk and supported the multidisciplinary decision to transition to comfort care.

Existing literature consistently supports early surgical management in cases of IE with periannular complications (Table 2). Graupner et al. and Miro et al. reported improved outcomes in patients undergoing timely surgical intervention for abscesses compared to conservative therapy [5,8]. The 2015 ESC guidelines also recommend urgent surgery in patients with heart failure, abscess formation, or persistent infection despite appropriate antibiotic therapy [7].

**Table 2:** Literature summary on aortic root abscess in mitral valve IE ESC: European Society of Cardiology, IE: Infective endocarditis

Author, Year	Case Details	Surgical Management	Outcome Summary
Anguera et al., 2005	Surgical case series; 1% of aortic root abscesses originated from mitral valve IE	Surgery performed in most cases; early intervention improved survival	Early surgical intervention associated with better outcomes
Graupner et al., 2002	Prospective analysis of periannular complications; abscess formation linked to increased mortality	Surgery recommended in presence of abscess or fistula	Non-surgical management resulted in higher mortality
Miro et al., 1999	Study of IE with aorto-mitral curtain involvement; majority had prior valve surgery or prosthetic devices	Surgical management common in prosthetic IE with complications	Delayed surgery associated with worse prognosis in high-risk patients
Habib et al., 2015 (ESC Guidelines)	Guidelines emphasize early surgery for IE with abscess, heart failure, or persistent infection	Urgent surgery advised for periannular complications	Strong evidence supports improved outcomes with early surgery

This case highlights the importance of considering ARA even in mitral valve IE, particularly in patients with prior valve repair. Diagnostic imaging can be limited in such patients, and surgical options may be constrained by comorbidities. Clinicians should maintain a high index of suspicion for periannular extension, pursue early imaging when feasible, and involve surgical teams promptly to optimize outcomes.

## Conclusions

Infective endocarditis complicated by aortic root abscess is a rare but life-threatening condition that requires high clinical suspicion, prompt imaging, aggressive medical therapy, and timely surgical intervention when feasible. However, clinical decisions regarding surgery can be challenging in patients with significant comorbidities and hemodynamic instability, as seen in this case. While early surgical intervention is often associated with improved outcomes, it may not be immediately possible in critically ill patients. This highlights the importance of early multidisciplinary evaluation and close reassessment to balance operative risk against disease progression in high-risk individuals.
